# Identification of an Autophagy-Related lncRNA Prognostic Signature and Related Tumor Immunity Research in Lung Adenocarcinoma

**DOI:** 10.3389/fgene.2021.767694

**Published:** 2021-12-08

**Authors:** Hang Chen, Zeyang Hu, Menglu Sang, Saiqi Ni, Yao Lin, Chengfang Wu, Yinyu Mu, Kaitai Liu, Shibo Wu, Ni Li, Guodong Xu

**Affiliations:** ^1^ Medical School, Ningbo University, Ningbo, China; ^2^ Department of Cardiothoracic Surgery, The Affiliated Lihuili Hospital, Ningbo University, Ningbo, China; ^3^ Department of Urology, Ningbo City First Hospital, Ningbo, China

**Keywords:** lung adenocarcinoma, long noncoding RNA, tumor immune microenvironment, prognostic signature, survival

## Abstract

Autophagy is closely associated with the tumor immune microenvironment (TIME) and prognosis of patients with lung adenocarcinoma (LUAD). In the present study, we established a signature on the basis of long noncoding RNAs (lncRNAs) related to autophagy (ARlncRNAs) to investigate the TIME and survival of patients with LUAD. We selected ARlncRNAs associated with prognosis to construct a model and divided each sample into different groups on the basis of risk score. The ARlncRNA signature could be recognized as an independent prognostic factor for patients with LUAD, and patients in the low-risk group had a greater survival advantage. Kyoto Encyclopedia of Genes and Genomes and Gene Ontology enrichment analysis suggested that several immune functions and pathways were enriched in different groups. A high-risk score correlated significantly negatively with high abundance of immune cells and stromal cells around the tumor and high tumor mutational burden. Low-risk patients had a higher PD-1, CTLA-4, and HAVCR2 expression and had a better efficacy of immune checkpoint inhibitors, including PD-1/CTLA-4 inhibitor. A reliable signature on the basis of ARlncRNAs was constructed to explore the TIME and prognosis of patients with LUAD, which could provide valuable information for individualized LUAD treatment.

## Introduction

Lung cancer is one of the malignant tumors with the highest morbidity and mortality in the world (Nasim et al., 2019). The incidence and mortality of lung cancer in the United States in 2021 are estimated to be 235,760 and 131,880, respectively (Siegel et al., 2021). In China, there were estimated 733,000 new lung cancer cases and 610,000 deaths in 2015 (Chen et al., 2016). Non–small cell lung cancer (NSCLC) accounts for about 85% of lung cancers, of which adenocarcinoma accounts for about 50% of NSCLC (Shi et al., 2019). Although the advent of radiotherapy and chemotherapy has revolutionized the NSCLC treatment, the 5-year survival rate of NSCLC with distant metastasis is only 7% (American Cancer Society, 2021). Therefore, it is crucial to screen a reliable biomarker to guide individualized treatment of NSCLC.

As a crucial part of the recycling process in complicated tumor immune microenvironment (TIME), autophagy in tumor immunity has been increasingly appreciated (Li et al., 2015). For example, Jiang et al. illustrated the impact of autophagy on TIME from three perspectives and proposed that autophagy-based therapy combined with immunotherapy may be promising (Jiang et al., 2021). Gerada and Ryan (2020) explained that TIME determines whether autophagy promotes or inhibits tumors. Long noncoding RNAs (LncRNAs) are a class of RNA that do not code proteins with transcripts >200 nucleotides (Cai et al., 2020). They participated in the progression and metastasis of lung adenocarcinoma (LUAD) and were associated with immune pathways and even served as a biomarker for prognosis of LUAD (Wei et al., 2017; Pang et al., 2021). Recently, the prognostic signatures on the basis of coding or noncoding genes to predict the prognosis of patients with LUAD have been a research hot spot (Zhang et al., 2021a; Li et al., 2021a). However, utilizing lncRNAs related to autophagy (ARlncRNAs) to construct models and exploring the tumor immunity and the efficacy of immunotherapy in patients with LUAD were still lacking. In this study, we established a novel ARlncRNA signature to analyze the TIME and prognosis of patients with LUAD, which represented a step toward individualized immunotherapy in LUAD.

## Materials and Methods

### Data Acquisition

**FIGURE 1:**
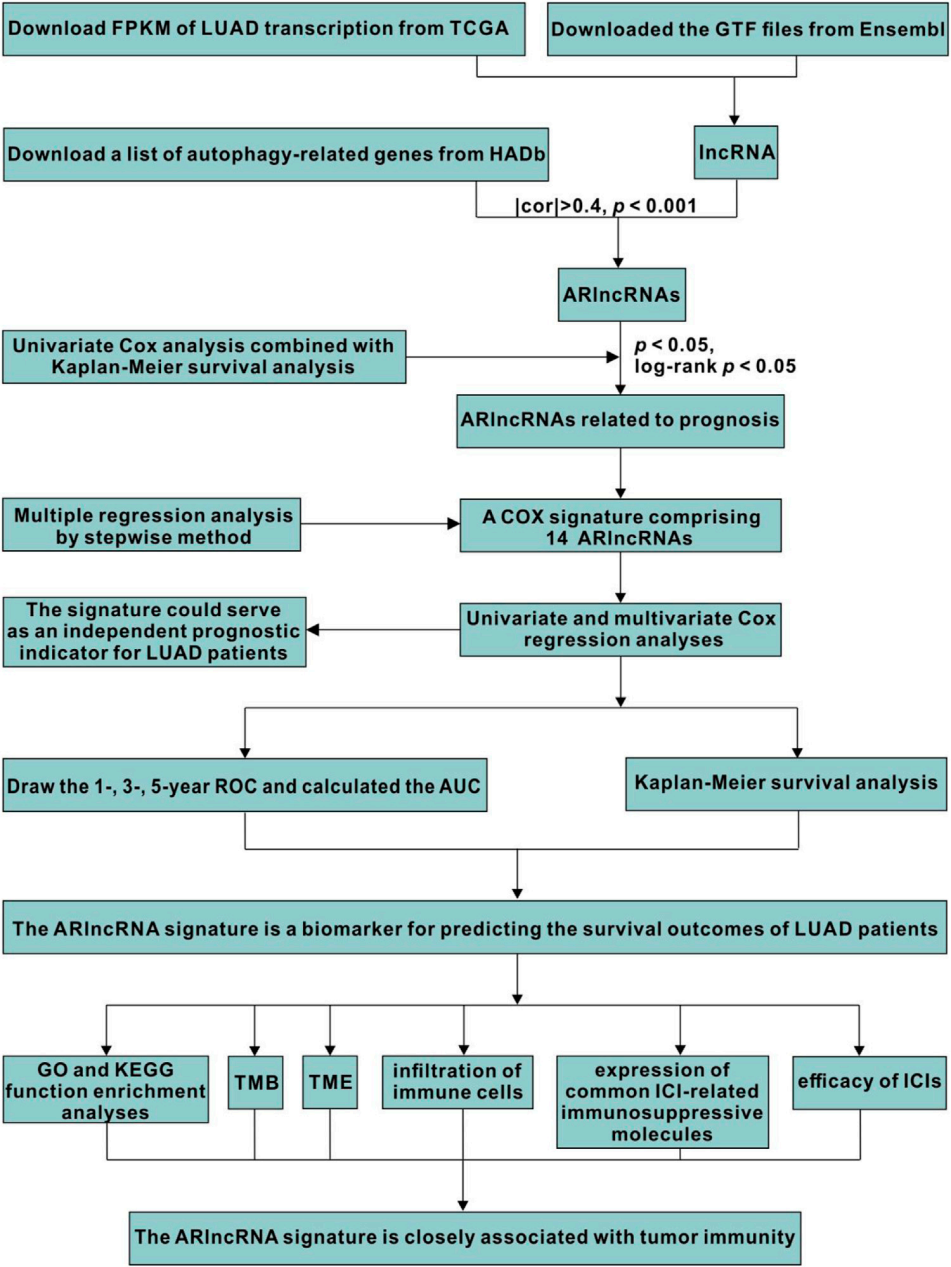
The flowchart for identifying a novel reliable ARlncRNA signature and its implication.

A signature on the basis of ARlncRNAs was established by a multi-step approach (Figure 1). We acquired the transcriptome profiles and the corresponding clinical information of patients with LUAD and normal samples from The Cancer Genome Atlas (TCGA, https://portal.gdc.cancer.gov/) (Tomczak et al., 2015) database in December 2020. The gene transfer format (GTF) files were obtained from Ensembl (http://asia.ensembl.org) (Howe et al., 2021) to distinguish lncRNAs and messenger RNAs (mRNAs). Furthermore, a list of autophagy-related genes (ARGs) from the Human Autophagy Database (HADb) (http://www.autophagy.lu/) was acquired. We identified ARlncRNAs by calculating the correlation coefficient between the ARGs and lncRNAs (|cor| > 0.4 and *p* < 0.001).

### Construction of the ARlncRNA Signature

Kaplan–Meier survival analysis combined with univariate Cox analysis was performed to screen the ARlncRNAs expression levels that were significantly associated with the overall survival (OS) of the patients with LUAD (log-rank *p* < 0.05, and *p* < 0.05). They were chosen for subsequent Cox proportional hazard regression analysis, and a heatmap was plotted for visualization. Subsequently, the multivariate Cox regression analysis was performed to construct the prognostic signature by running the R-x64-4.0.4 survival package, and the risk score of every patient with LUAD was calculated on the basis of the following formula:

$$\begin{array}{l} \;\;\;\;\;\;\;\;\;\;\;\;\;\;\;\;\;\;\;\;\;14\\ \mathrm{Risk}\;\mathrm{score}=\sum \mathrm{Coef}\left(i\right)\times E\left(i\right)\\ \;\;\;\;\;\;\;\;\;\;\;\;\;\;\;\;\;\;\;\;\;\mathrm{i=1}\; \end{array}$$

Coef(i) (Table 1) and E(i) represent the regression coefficient of the multivariate Cox analysis for each ARlncRNA and each ARlncRNA expression level, respectively. The median value of risk score was considered as the cutoff point for differentiating each patient into different groups.

**TABLE 1:** The regression coefficient of each ARlncRNA included by the multivariate Cox analysis.

ID	Coef	HR
AC093673.1	0.031656013	1.032162394
CARD8-AS1	−0.144812079	0.865184864
AC060780.1	0.182095366	1.199728602
AC123595.1	−0.268233505	0.764729192
UGDH-AS1	−0.380154182	0.683755978
LINC00996	−0.392113955	0.675627119
LINC00861	0.31943108	1.376344511
AF131215.5	−0.207575707	0.812551724
AC008763.1	−0.234324141	0.791105345
AL136304.1	−0.266108078	0.766356297
AL606489.1	0.238386541	1.269199696
HLA-DQB1-AS1	−0.04503729	0.955961834
LINC00654	−0.195989673	0.822020724
LINC00847	−0.025910346	0.974422446

### Validation of the Risk Prognosis Model

Several receiver operating characteristic (ROC) curves were generated, and area under the ROC curve (AUC) was calculated by R-x64-4.0.4 survival, survminer, and timeROC packages to validate the predictive value of the prognostic signature. Kaplan–Meier log-rank test was conducted for comparing the OS between different risk groups to assess the predictive value of the prognosis model. A barplot and a boxplot were generated by R-x64-4.0.4 packages plyr, ggplot2, and ggpubr to study whether there are statistical differences in OS between different risk groups. In addition, univariate and multivariate Cox regression analyses were performed to explore whether the prognostic signature was a potential independent prognostic indicator for patients with LUAD, and the results were visualized with two forest maps. To study the correlation between risk score and clinicopathological characteristics, Wilcoxon rank sum tests were performed to plot boxplots. A heatmap and multiple Kaplan–Meier survival analyses were generated to visualize the expression and prognosis of ARlncRNAs included in the process of modeling, respectively.

### Enrichment of Functions and Pathways in the ARlncRNA Signature

Differentially expressed genes between different risk groups were identified by performing differential expression analysis. The R-x64-4.0.4 packages limma was utilized, and the significance threshold for determining differentially expressed genes was log fold change > 1 and false discovery rate < 0.05. To filter functional phenotypes in different risk groups, we performed gene set enrichment analysis (GSEA) 4.0.1 (https://www.gsea-msigdb.org/gsea/index.jsp) (Subramanian et al., 2005) by Kyoto Encyclopedia of Genes and Genomes (KEGG) (*p* < 0.05 and *q* < 0.25). Gene Ontology (GO) function enrichment analysis was conducted to study the functional phenotypes where differentially expressed ARlncRNAs were enriched (*p* < 0.05 and *q* < 0.05).

### Correlation Analysis of Tumor Mutational Burden

Spearman analysis was conducted to calculate the correlation coefficient between the tumor mutational burden (TMB) and the constructed model on the basis of R-x64-4.0.4 ggplot2, ggpubr, and ggExtra packages. The differences in TMB between different risk groups were explored by Wilcoxon signed-rank test, and a boxplot was plotted. To study the connection between TMB and the prognostic signature comprehensively, Kaplan–Meier survival analyses were performed for comparing OS between high TMB and low TMB.

### Estimation of Tumor Immune Microenvironment of the Prognostic Signature

To explore the abundance of immune cells and stromal cells between different groups, StromalScore, ImmuneScore, and ESTIMATEScore (StromalScore + ImmuneScore) of each patient were calculated by R-x64-4.0.4 estimate and limma packages. Then, Wilcoxon rank sum tests were utilized to explore the differences in StromalScore, ImmuneScore, and ESTIMATEScore between different groups, and three column diagrams were plotted for visualization. Single-sample GSEA (ssGSEA) was conducted for scoring LUAD-infiltrating immune cells to quantify their relative content. The scores of immune cells and pathways in different groups were shown on multi-boxplots, respectively.

### Evaluation of Clinical Immunotherapy Efficacy of ARlncRNA Signature

The emergence of immunotherapy has revolutionized the treatment of NSCLC. National Comprehensive Cancer Network (NCCN) guidelines (National Comprehensive Cancer Network, 2021) recommend a series of immunotherapy drugs, including CTLA-4 and PD-1 blocking antibodies for NSCLC treatment. Expression analyses of common immune checkpoint inhibitor (ICI)–related immunosuppressive molecules (e.g., PD-1, CTLA-4, and HAVCR-2) were conducted by R-x64-4.0.3, ggpubr, and limma packages to detect any statistical differences in expression of common ICI-related immunosuppressive molecules between different risk groups, and several violin plots were generated for visualization. Immunophenoscore (IPS) representing the immunotherapy effect of patients with LUAD was extracted and obtained from the The Cancer Immunome Atlas (https://tcia.at/) database (Hugo et al., 2017). The difference in immunotherapy efficacy for patients with LUAD between different groups was calculated by Wilcoxon rank sum tests, and boxplots were labeled for visualization as follows: ***<0.001, **<0.01, and *<0.05.

## Results

### Identification of ARlncRNAs

Figure 1 shows a multi-step approach that was conducted to identify ARlncRNAs. A total of 551 LUAD transcriptome data, corresponding clinical data from TCGA-LUAD cohort (54 normal samples and 497 LUAD samples), and 232 ARGs from HADb were downloaded. Subsequently, we distinguished the mRNAs and lncRNAs by GTF files and identified 1,071 ARlncRNAs on the basis of Spearman correlation analysis.

### Establishment of the Prognostic Signature Based on ARlncRNAs

**FIGURE 2:**
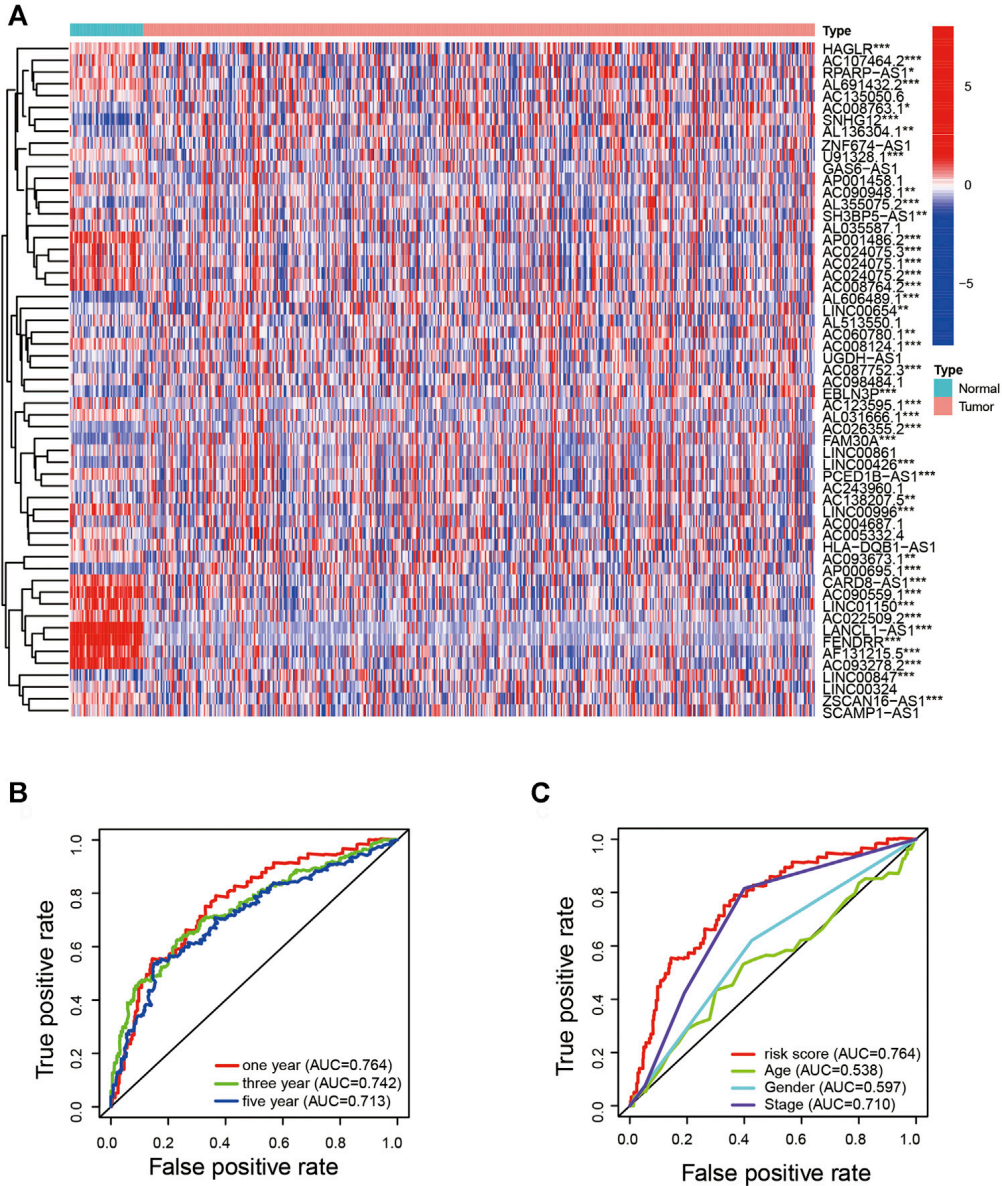
Identification of the ARlncRNA signature. **(A)** Heatmap shows the expression of ARlncRNAs related to survival in LUAD and normal samples. **(B)** The 1-, 3-, and 5-year AUC values were >0.7. **(C)** Compared with AUC of age, sex, and stage, the 1-year AUC value was the maximum.

Using univariate Cox analysis combined with Kaplan–Meier survival analysis, we screened out 57 ARlncRNAs associated with survival of patients with LUAD (Figure 2A). Multivariate Cox regression analysis yielded 14 ARlncRNAs among ARlncRNAs related to prognosis for subsequent modeling. The risk score of every patient was calculated on the basis of correlation coefficients calculated by multivariate Cox regression analysis, and each sample was differentiated into different groups by median value of risk score.

### The Prognostic Signature Is a Powerful LUAD Prognostic Indicator

**FIGURE 3:**
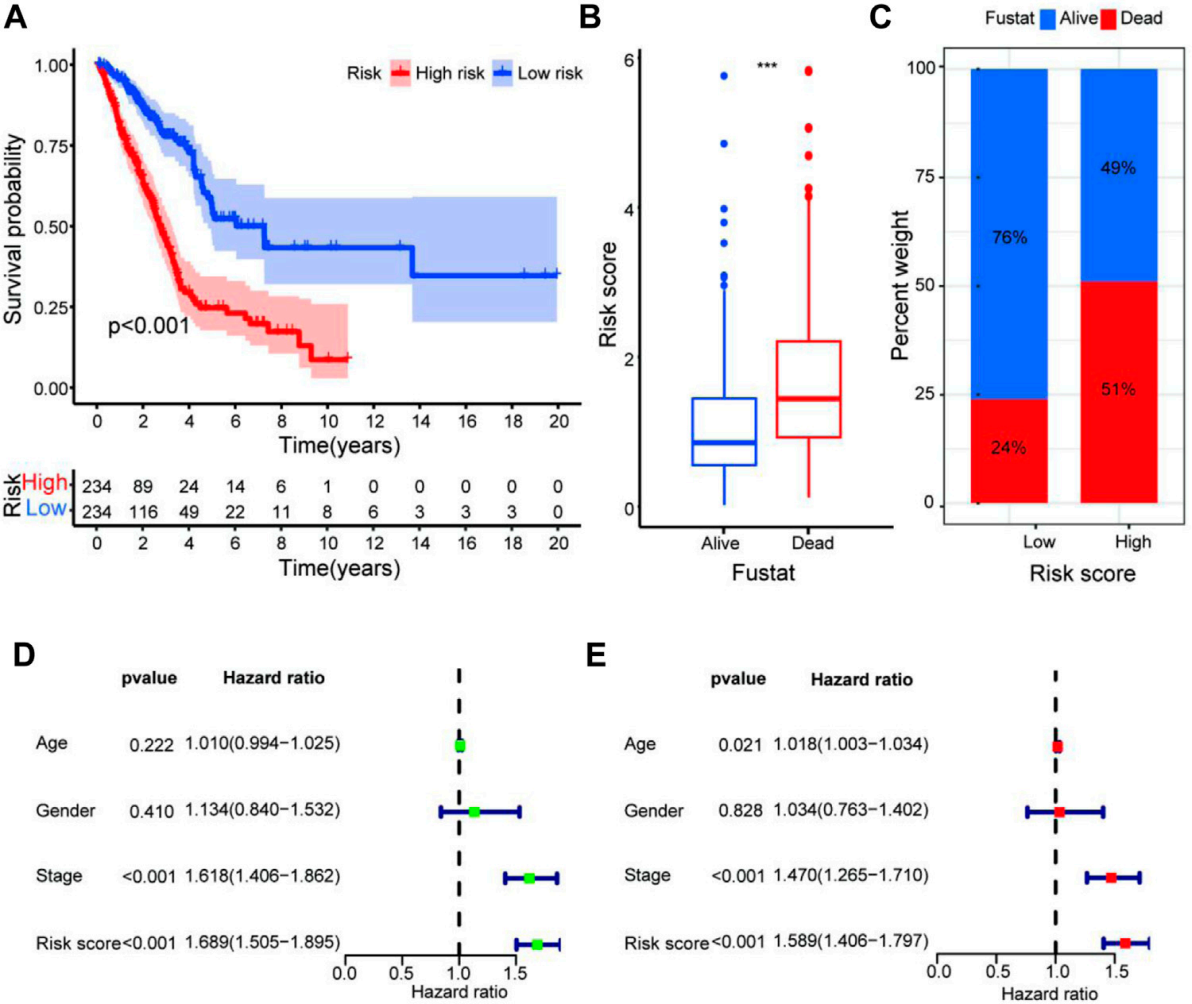
Validation of the predictive value of the prognostic signature. **(A–C)** The survival curve **(A)**, boxplot **(B)**, and barplot **(C)** indicate that the ARlncRNA signature was associated with survival outcomes of patients with LUAD, and patients with low risk have a significant survival advantage. **(D,E)** Univariate **(D)** and multivariate **(E)** Cox regression analyses including age, gender, stage, and risk score suggest that risk score (HR = 1.589, CI = 1.406–1.797, *p* < 0.001) and stage (HR = 1.470, CI = 1.265–1.710, *p* < 0.001) could act as an independent prognostic factor for LUAD.

**FIGURE 4:**
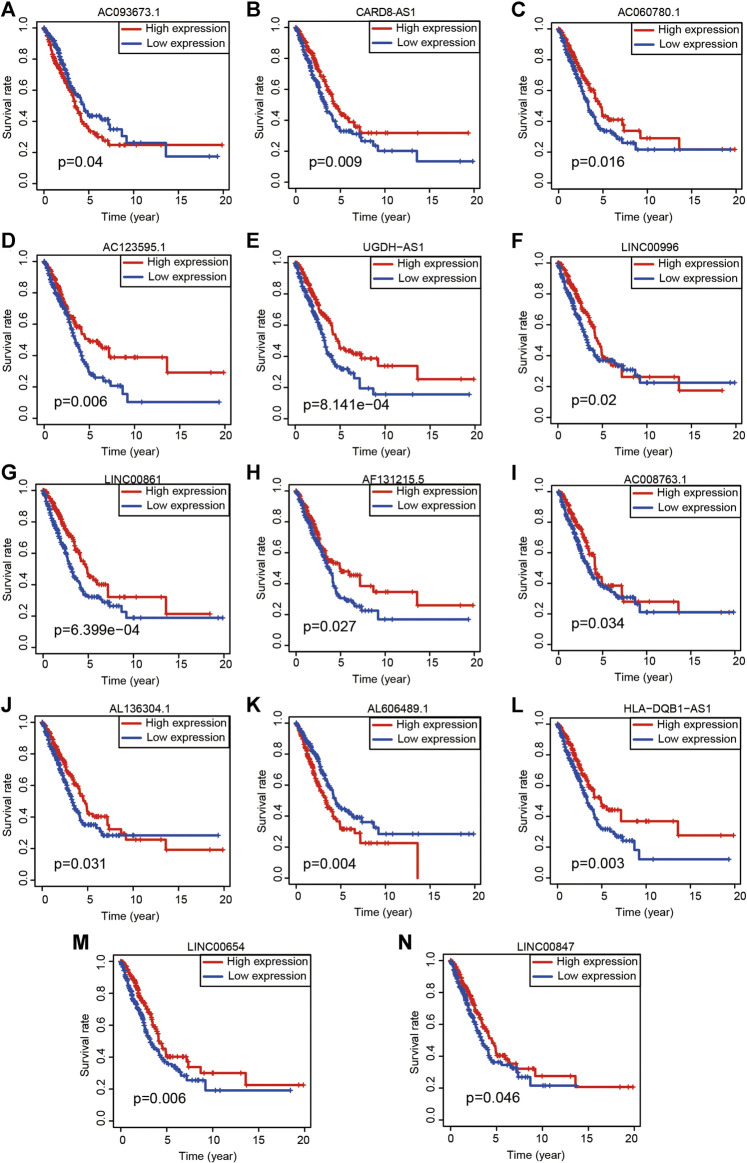
Kaplan-Meier survival analyses of 14 ARlncRNAs included in the modeling process. (**A–N**) The survival curves of 14 ARlncRNAs included by the multivariate Cox regression.

**FIGURE 5:**
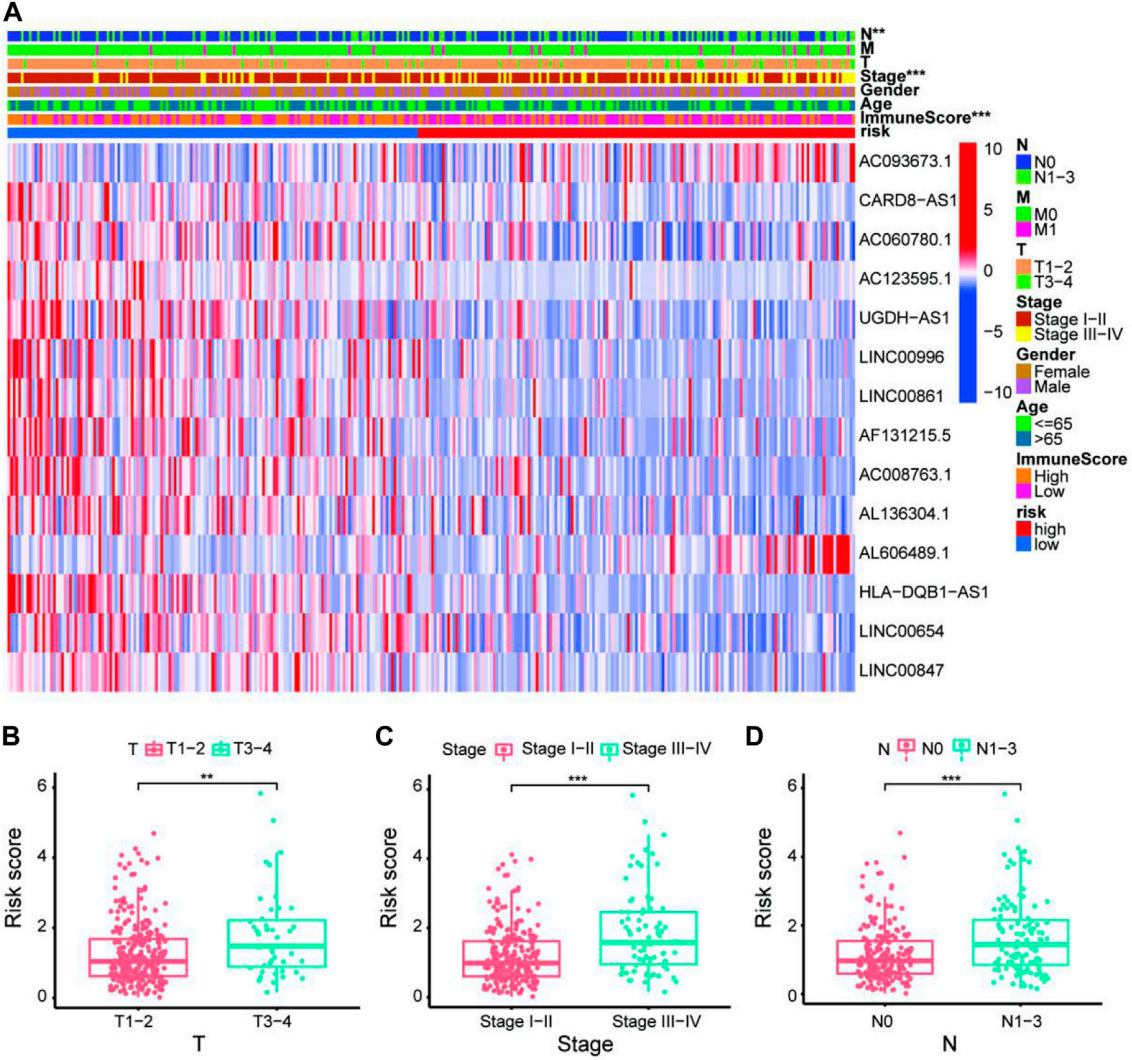
Exploration of clinicopathological characteristics of the 14-ARlncRNA signature. **(A)** The heatmap suggested that stage (*p* < 0.001), ImmuneScore (*p* < 0.001), and N (*p* < 0.01) were statistically different in different groups. In addition, most ARlncRNAs included in the modeling process were enriched in the low-risk group, suggesting that autophagy was more active in the low-risk group. **(B–D)** The scatter diagrams indicated that patients with LUAD with advanced T, advanced stage, or advanced N tended to have higher-risk score and statistically significant.

To quantify the predictive ability of the ARlncRNA signature, we generated several ROC for validation. The curves demonstrated that 1-, 3-, and 5-year AUC values were 0.764, 0.742, and 0.713, respectively (Figure 2B). Furthermore, compared with other clinicopathological characteristics (e.g., age, gender, and stage), the 1-year AUC value was the maximum (Figure 2C). The survival curve, boxplot, and barplot indicated that patients with low risk had a better prognosis and were statistically significant (Figures 3A–C). To explore the relationship between the ARlncRNA signature with clinicopathological characteristics, we performed univariate and multivariate Cox regression analysis, which suggested that two factors, i.e., the risk score [hazard ratio (HR) = 1.589, confidence interval (CI) = 1.406–1.797, *p* < 0.001] and stage (HR = 1.470, CI = 1.265–1.710, *p* < 0.001), correlated with the survival (Figures 3D,E). The 14 ARlncRNAs included in the modeling process were closely associated with survival outcomes of patients with LUAD (Figures 4A–N). The results above suggested that the ARlncRNA signature could act as a potential independent prognostic indicator for patients with LUAD. The heatmap (Figure 5A) suggested that stage (*p* < 0.001) and ImmuneScore (*p* < 0.001), N (*p* < 0.01) were statistically different in different groups. In addition, most ARlncRNAs included in the process of modeling were enriched in the low-risk group, suggesting that autophagy was more active in the low-risk group. The boxplots indicated that patients with LUAD with advanced T (Figure 5B, T_3-4_, *p* < 0.01), advanced stage (Figure 5C, Stage III-IV, *p* < 0.001), or advanced N (Figure 5D, N_1-3,_ *p* < 0.001) tended to have higher-risk score and statistically significant.

### Functional Annotation of the Prognostic Signature

**FIGURE 6:**
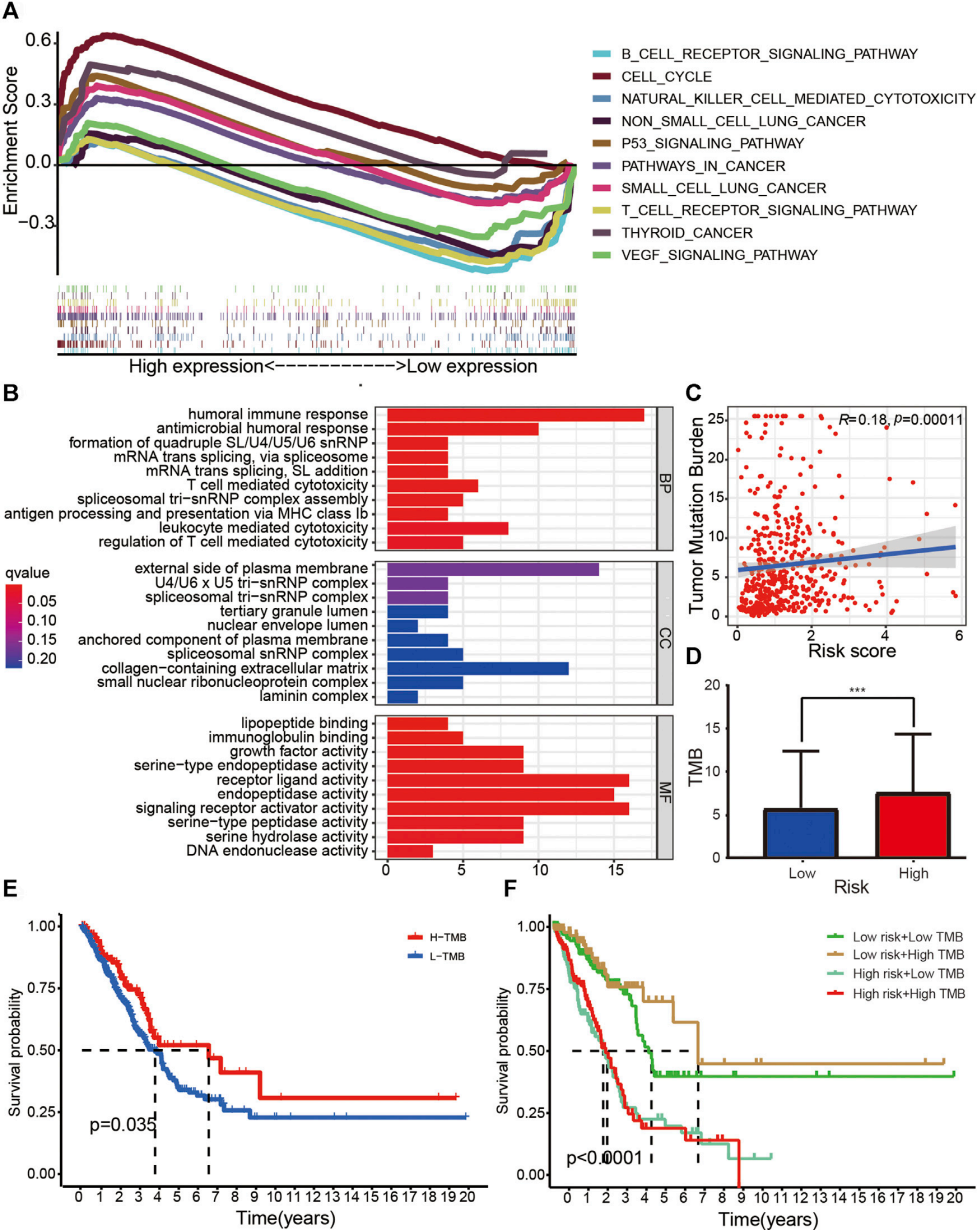
Research on tumor immunity of the 14-ARlncRNA signature. **(A,B)** KEGG **(A)** and GO **(B)** function enrichment analyses reveal that several immune pathways and immune function were enriched in different groups, suggesting the ARlncRNA signature may be associated with tumor immunity. **(C,D)** The scatter diagram **(C)** and column diagram **(D)** show that high-risk patients exhibit significantly higher TMB. **(E,F)** The survival curves indicate that patients with a combination of low-risk and high TMB showed a greater prognosis.

GSEA (Figure 6A) indicated that several immune pathways, such as B cell receptor signaling, natural killer cell–mediated cytotoxicity, T cell receptor signaling pathway, and vascular endothelial growth factor (VEGF) signaling pathway, were enriched in patients with low risk. Cell cycle, P53 signaling pathway, pathways in cancer, small cell lung cancer, and thyroid cancer were relatively more active in the high-risk group. GO function enrichment analysis indicated that several functions related to tumor immunity, such as humoral immune response, antimicrobial humoral response, antigen processing and presentation, and T cell–mediated cytotoxicity, were enriched in different groups (Figure 6B).

### Assessment of the Correlation Between the TMB and ARlncRNA Signature

The correlation curve (Figure 6C) and boxplots (Figure 6D) indicated that TMB was significantly negatively correlated with risk score, and the TMB of patients with high risk was significantly higher than those with low risk (*p* < 0.001). According to survival curves (Figures 6E,F), patients with high TMB had a better survival advantage (*p* = 0.035), and patients with a combination of low risk and high TMB showed a great prognosis.

### Tumor Immune Microenvironment of the ARlncRNA Signature

**FIGURE 7:**
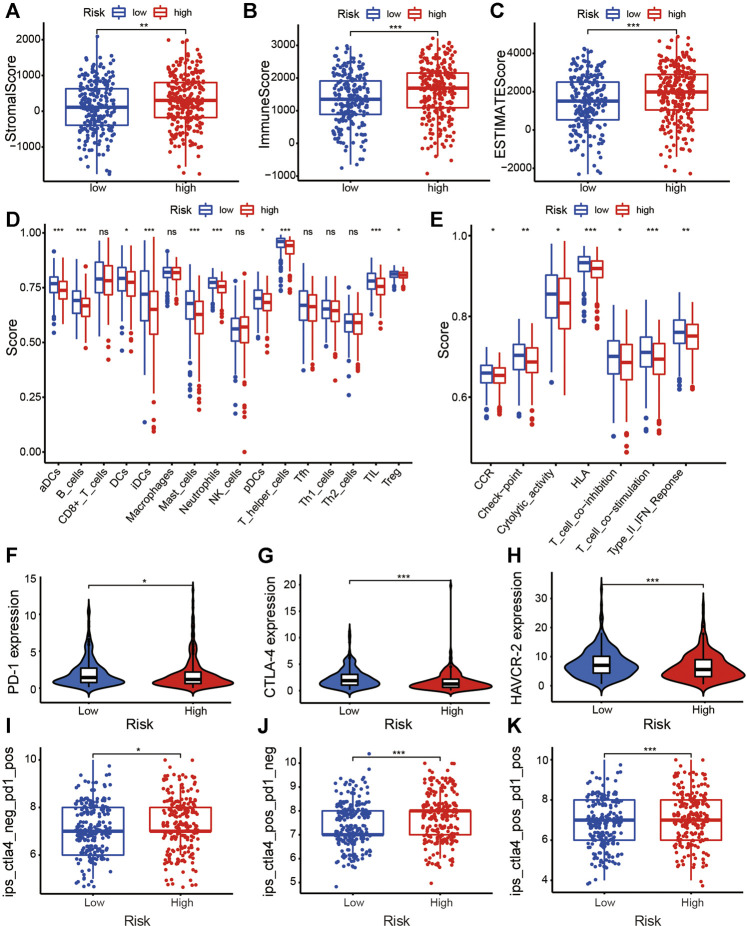
Exploration of the tumor immunity and ICI efficacy of the ARlncRNA signature. **(A–C)** The column diagrams reveal that low-risk group patients have a higher StromalScore **(A)**, ImmuneScore **(B)**, and ESTIMATEScore **(C)**. **(D,E)** The multi-boxplots show that patients in low-risk group have a higher abundance of aDCs, B cells, DCs, iDCs, mast cells, neutrophils, pDCs, T helper cells, TILs, and Tregs **(D)**. Several immune pathways, including CCR, checkpoint, cytolytic activity, HLA, T cell co-inhibition, T cell co-stimulation, and type II IFN response were enriched in low-risk patients **(E)**. **(F–H)** The violin plots indicate that high-risk score correlated significantly negatively with high PD-1 **(F)**, CTLA-4 **(G)**, and HAVCR-2 **(H)** expression, revealing low-risk patients are more applicable for ICIs treatment. **(I–K)** The violin plots validate the results above that regardless of whether it is a PD-1 blocker alone **(I)**, a CTLA-4 blocker alone **(J)**, or a combination of the two **(K)**, the efficacy of patients with low-risk is better than that of high-risk patients.

We found that StromalScore (Figure 7A, *p* < 0.001), ImmuneScore (Figure 7B, *p* < 0.001), and ESTIMATEScore (Figure 7C, *p* < 0.001) in low-risk patients were significantly higher than that of high-risk patients. The multi-boxplots (Figures 7D,E) indicated that the abundance of activated dendritic cells (aDCs), B cells, dendritic cells (DCs), immature dendritic cells (iDCs), mast cells, neutrophils, plasmacytoid dendritic cells (pDCs), T helper cells, tumor infiltrating lymphocytes (TILs), and regulatory T cells (Tregs) associated significantly negatively with the risk score. Compared with the high-risk group, several immune pathways, e.g., clinical complete response (CCR), checkpoint, cytolytic activity, human leukocyte antigen (HLA), T cell co-inhibition, T cell co-stimulation, and type II interferon (type II IFN) response were more active in the low-risk group.

### ARlncRNA Signature in the Role of Immunotherapy

The violin plots showed that the expression of common ICI-related immunosuppressive molecules, e.g., PD-1 (Figure 7F, *p* < 0.05), CTLA-4 (Figure 7G, *p* < 0.001), and HAVCR-2 (Figure 7H, *p* < 0.001), in low-risk group was significantly higher than those in high-risk group, which indicated that the efficacy of the abovementioned ICIs seem to be better for low-risk patients. The boxplots on the basis of IPS validated the above results, suggesting that, whether it is PD-1 inhibitor alone (Figure 7I, *p* < 0.05), CTLA-4 inhibitor alone (Figure 7J, *p* < 0.001), or a combination of the two (Figure 7K, *p* < 0.001), the efficacy of low-risk patients is better than that of high-risk patients.

## Discussion

Recently, the prognostic signatures on the basis of bioinformatics analyses for forecasting the survival outcome of cancer patients have become more and more popular (Liu et al., 2021a; Zhang et al., 2021b). Although there are already lots of signatures utilizing ARlncRNAs (Wu et al., 2021a) or ARGs (Duan et al., 2021) to predict the survival outcomes of patients with LUAS, we are the first to explore the tumor immunity of the ARlncRNA model in detail. We conducted a detailed study on the tumor immunity of the ARlncRNA signature, which renders the constructed signature applicable for guiding the clinical personalized treatment of patients with LUAD.

First, the lncRNA and ARG transcriptome profiles were obtained, and ARlncRNAs related to prognosis of patients with LUAD on the basis of co-expression analysis and univariate Cox analysis were identified. Next, we calculated the 1-, 3-, and 5-year AUC values to obtain an ideal signature and differentiated each patient into different groups on the basis of median value. Then, the survival and clinicopathological characteristics were analyzed to assess the predictive value of the ARlncRNA signature. Subsequently, we conducted a comprehensive assessment of the tumor immunity of the ARlncRNA signature, including GO and KEGG function enrichment analyses, TMB, TME, infiltration of immune cells, expression of common ICI-related immunosuppressive molecules, and efficacy of ICIs.

Previous studies on LUAD have mostly focused on single genes or noncoding genes, which are unable to illustrate the complex tumorigenesis and development process (Fan et al., 2018; Wang et al., 2020). In recent years, a combination of several genes to improve the predictive value of OS in patients with LUAD was gradually identified. For example, Duan et al. (2021) constructed a prognostic signature on the basis of ARGs, which served as a novel biomarker in LUAD. Meanwhile, Zhu et al. (2021) identified a ferroptosis-related gene signature and explored the immune cells infiltration. In this study, several ARlncRNAs included in the modeling process have been already reported in various malignant tumors, such as CARD8-AS1 (Lin et al., 2018; Li and Zhan, 2019), AC060780.1 (Curty et al., 2020), AC123595.1 (Li et al., 2020; Zheng et al., 2020), UGDH-AS1 (Liu et al., 2020), LINC00996 (Ge et al., 2018), LINC00861 (Qian et al., 2018; Liu et al., 2021b; Hu et al., 2021), AL606489.1 (Wu et al., 2021a), HLA-DQB1-AS1 (Singh and Gasman, 2020), LINC00654 (Li et al., 2017), and LINC00847 (Tu et al., 2020; Li et al., 2021b). Whereas, others have not been discovered yet and may be potential novel biomarkers for further study.

Researchers found that autophagy played a vital role in tumorigenesis and development. Gu et al. confirmed that bupivacaine-induced autophagy through Akt/mTOR signaling, inhibiting the progression of NSCLC (Gu et al., 2021). Lin et al. proposed that high expression of miR-30a improved the prognosis of NSCLC after neoadjuvant chemotherapy by reducing autophagy caused by chemotherapy drugs (Lin et al., 2021). In addition, Li et al. found that the dysfunction of autophagy mediated by c-myc/miR-150/EPG5 had a great impact on the progression of NSCLC (Li et al., 2019). Collectively, autophagy was probably involved in the occurrence and development of LUAD through a certain signaling pathway, having a significant impact on the prognosis of patients with LUAD.

Then, GSEA enrichment analysis suggested that patients with low risk had more autophagy and were enriched in B cell receptor signaling, natural killer cell–mediated cytotoxicity, T cell receptor signaling pathway, and the VEGF signaling pathway. Recent searches confirmed the strong correlation between autophagy and VEGF. For example, Chen et al. found that VEGF promoted the occurrence of autophagy and VEGF knockdown decreased the autophagy level (Chen et al., 2020). In addition, Spengler et al. (2020) discovered that VEGF signaling pathway regulated autophagy in endothelial cells. Together, we speculated that autophagy probably contributed to the occurrence and development of LUAD through VEGF signaling pathway and that the ARlncRNA signature was closely associated with tumor immunity.

Recently, the interaction between autophagy and tumor immunity was investigated comprehensively. For example, TMB and TIME were identified as important determinants of the efficacy of ICIs and in the prognosis of cancer patients (Wu et al., 2021b). Moreover, Jena et al. (2021) have demonstrated that autophagy was closely related to TIME and participated in tumor progression. To explore the relationship between the ARlncRNA signature and tumor immunity thoroughly, we conducted ssGSEA to investigate the immune status in different groups. The patients in low-risk group had a higher abundance of immune cells and were more active in immune pathways, most of which were validated closely associated with autophagy. For example, Di Rita and Strappazzon (2021) found that CALCOCO2, an autophagy receptor, was mainly expressed in B cells, which mediated autophagy. In addition, the function of DCs to secrete cytokines has been shown to be inhibited by autophagy (Liang et al., 2021). Turan et al. (2019) proved through experiments that iDCs infected with TSV-1 could induce autophagy. Li et al. (2021c) demonstrated that autophagy of mast cells could serve as a therapeutic target for allergic reactions. Ding et al. (2021) revealed that neutrophils were associated with autophagy. Autophagy has been validated to fuel pDCs (Basit et al., 2018). Schmid et al. (2006) proposed that CD4(+) T helper cells could recognize MHCII molecules presented after autophagy. Lu et al. (2021) demonstrated that autophagy could mediate the function of Tregs. Samuel et al. (2013) proved that cellular metabolism facilitated autophagy to mediate the cytolytic effect. Zhang et al. (2020) confirmed the firm correlation between autophagy and HLA. These studies above suggest that autophagy is closely linked to tumor immunity, and autophagy probably participates in LUAD progression by regulating tumor immunity.

At present, PD-1 (Fan et al., 2021) and CTLA-4 (Sun et al., 2020) inhibitors have been validated to benefit patients with advanced NSCLC in clinical trials. Furthermore, the research indicated that the autophagy of tumor cells increased the expression of ICI-related immunosuppressive molecules (e.g., PD-1 and CTLA-4) and affected anti-tumor immune responses directly. In this study, expression analyses were conducted to study the correlation between the ARlncRNA signature and the expression of common ICI-related immunosuppressive molecules, which revealed that low-risk patients always had a higher expression of them and a better efficacy of ICIs. Subsequently, we analyzed the efficacy of ICIs and verified the results above, demonstrating that regardless of whether it is a PD-1 inhibitor alone, a CTLA-4 inhibitor alone, or a combination of the two, the efficacy of patients in the low-risk group is better than that of the high-risk patients. Overall, the ARlncRNA signature could serve as a novel indicator for screening patients applicable for ICIs.

According to our data, we speculated that compared with high-risk patients, low-risk patients have more active autophagy, stronger tumor immunity, and greater survival advantage and are more applicable for ICIs treatment. Autophagy could play a crucial part in the progression of LUAD by regulating the tumor immunity through VEGF signaling pathway, which had a great impact on prognosis of patients with LUAD.

However, there are several limitations in our research. First, it is the bias of the information in the analysis process, because the profiles were obtained from public database. Second, it was difficult to find an ideal Gene Expression Omnibus set including both 14 lncRNAs newly identified and detailed clinical information to validate the constructed ARlncRNA signature. Last, external validation, such as quantitative real-time PCR and microarrays are necessary to increase credibility.

In conclusion, we constructed a novel ARlncRNA signature and predicted the survival of patients with LUAD, the state of TIME, and even the efficacy of ICIs accurately on the basis of the expression of the 14 ARlncRNAs included in the modeling process, which may benefit patients with advanced NSCLC. Immunotherapy combined with TIME-targeted therapy may improve individualized treatment of LUAD in the future.

## Data Availability

The original contributions presented in the study are included in the article/Supplementary Material; further inquiries can be directed to the corresponding author.
